# Serum DHEA-S levels could be used as a comparable diagnostic test to assess the pubertal growth spurt in dentofacial orthopedics

**DOI:** 10.1186/s40510-020-00317-5

**Published:** 2020-06-22

**Authors:** V. Anusuya, Amit Nagar, Pradeep Tandon, G. K. Singh, Gyan Prakash Singh, A. A. Mahdi

**Affiliations:** 1 Department of Dentistry, All India Institute of Medical Sciences, Bhubaneswar, Odisha 751019 India; 2 Department of Orthodontics and Dentofacial Orthopaedics, King George’s Medical University, Lucknow, UttarPradesh 226003 India; 3 Department of Biochemistry, King George’s Medical University, Lucknow, Uttarpradesh 226003 India; 4 Tamilnadu, India

**Keywords:** Serum DHEA-S, IGF-1, CS stages, Skeletal growth, Cephalometrics, Growth assessment, Biomarker

## Abstract

**Background:**

Pubertal growth spurt assessment guides the timing of intervention for correcting the skeletal discrepancies in dentofacial orthopedics. Serum hormones are being studied for the skeletal age assessment to avoid unnecessary radiographic exposure. The present study is to evaluate the relationship of serum hormones dehydroepiandrosterone-sulfate (DHEA-S), insulin-like growth factor (IGF-1), and cervical vertebral stages (CS stages) in the skeletal age assessment of orthodontics patients around the circumpubertal age.

**Methods:**

A total of ninety subjects with age ranging from 7 to 21 years were selected and divided into two groups based on the sex (45 males, 45 females). They were further distributed in each group based on the six CS stages determined from the lateral cephalogram. Blood samples from each subject were collected to evaluate the serum DHEA-S and IGF-1 levels by using the enzyme-linked immunosorbent assay (ELISA). Collected data were analyzed in SPSS software with a test of normalcy, unpaired *t* test, and one-way analysis of variance (ANOVA) followed by the least significant difference (LSD) post hoc comparison test and univariate regression analysis.

**Results:**

The highest mean serum hormone levels were found in CS 4 in group A (male) and CS 3 in group B (female). ANOVA results showed that there was a significant difference in the serum hormone levels among the different CS stages in both the groups for both the hormones. Statistically, a significant difference was found between each CS stages for both the hormones except in the DHEA-S levels between CS 5 and CS 6.

**Conclusions:**

The mean serum DHEA-S levels followed a typical pattern from the CS 1 till CS 6 which was comparable and similar to the mean serum IGF-1 levels in respect to CS stages. Thus, serum DHEA-S levels could be used as a possible diagnostic test for the assessment of the skeletal pubertal growth spurt in dentofacial orthopedics.

## Background

The age of an individual is one of the important determinant factors in the treatment planning of malocclusion. It guides the orthodontist to allocate the growth-related treatment modalities in growing individuals. Among the different biological ages, skeletal age determination using hand-wrist radiographs is the gold standard for the assessment of overall skeletal growth status and the craniofacial structures [1]. Skeletal age reveals the present growth status as well as the remaining skeletal growth. In dentofacial orthopedics, treatment timing is mainly based on the pubertal growth spurts [2]. Skeletal class III malocclusion is best corrected during the pre-pubertal stage and skeletal class II during the pubertal growth spurt [3–6]. Thus, skeletal age determination is crucial to start the dentofacial interception at the right time to correct the skeletal deficiencies, in turn, to get the best possible treatment results [7].

Various tools are available for skeletal age determination, being gold standard; hand-wrist radiograph needs additional radiograph to be taken in the orthodontic patients [8–12]. Thus, cervical stages determined from the lateral cephalogram are routinely used for this purpose in patients undergoing orthodontic treatment [13]. Recently, biomarkers are evaluated to determine the circumpubertal growth stages. Hormones and bio-molecules evaluated from the serum, saliva, and urine were well reported to determine the skeletal age [14–17]. Serum IGF-1 has already been studied and established as a biomarker for the skeletal pubertal growth assessment [18, 19]. But as far as IGF-1 is concerned, the availability of laboratories for the testing and the cost are high. So, an alternative which could be easily available and cost-effective will be a better biomarker to use in day-to-day practice.

Puberty and pubertal growth spurt occur as a manifestation of changes and a delicate balance between various hormones, especially the growth and sex hormones [20]. It has been observed that the synchronized hormonal events take place even before the growth spurt in puberty. Adrenarche is one of the events, where there is an onset of production of DHEA and DHEA-S from the zona reticularis of the adrenal gland [21]. DHEA is the most abundant circulating steroid which gets sulfated into DHEA-S by sulfatases [22]. DHAE-S, an important factor in bone accretion, regulates bone mineral density and bone deposition during growth. In combination with carnitine, it promotes alkaline phosphatase activity and collagen I synthesis. Another function is to stimulate IGF-1 which in turn stimulates estrogen production that results in enhanced stimulation of the bone formation [23–25].

The role of DHEA-S in puberty, in the skeletal growth regulation, and also the DHEA-S diagnostic test which is more commonly used led us to this present study. It was to test the null hypothesis that there is no relationship exists between the serum DHEA-S levels and CS stages determined from the lateral cephalogram in the subjects of circum-pubertal age, in addition the relationship of IGF-1 and DHEA-S levels in different CS stages.

## Material and methods

The present study was conducted in patients with an age range between 7 to 21 years, with mean age of 13.65 ± 4.01 years, who were enrolled for orthodontic treatment in the Department of Orthodontics & Dentofacial Orthopedics in collaboration with the Department of Biochemistry, King George Medical University, Lucknow, Uttar Pradesh, India. All the subjects were apparently normal and healthy as per the Revised 2014 IAP Growth charts for height, weight, and body mass index (BMI) between 3rd and 97th percentiles of standards [26], which ensured good nutritional status and adequate growth velocity at the proper time. Subjects with a history of bleeding disorders, syndromes, chronic medication, hormone replacement therapy, and hormonal disorders and a history of trauma or surgery or anomaly in the area of cervical vertebrae were excluded from the study. Ethical approval was obtained from the institutional ethical committee (project no: 72nd ECM II-B-IMR/P2). After obtaining the written consent, demographic data were recorded individually for the subjects who fulfilled the inclusion and exclusion criteria.

The sample size was calculated, with 95% confidence interval, 80% power, 5% significance level, and less than 20 ng/ml of error of estimation by keeping the mean IGF-1 serum level of 298.6 ng/ml with a standard deviation of 85 ng/ml in cervical vertebrae maturation (CVM) stage 2 patients [18]. It was found that 69 is the minimum needed sample. But it was inflated as a total sample of 90 for the convenience of distribution in each group. Subjects were divided into 2 groups based on sex—group A (male) and group B (female), each consisting of 45 subjects. In each group, further distribution was done based on the six CS stages of the modified CVM stage given by Baccetti et al. [13].

Digital lateral cephalometric radiographs (Kodak T-mat Blue; Kodak Limited, Hemel Hempstead, Hertfordshire, UK) were recorded by positioning the subjects in a cephalostat at natural head position with the X-ray machine parameters kept at 8 mA and 80 kvp. Subjects were instructed to maintain the centric occlusion with lips at repose during the radiographic exposure. All the radiographs were recorded by the same technician. The radiographs were given random numbers after retrieval; thus, the examiner was blinded about the details of the subjects to avoid bias. The superior, inferior, posterior, and anterior borders of the second, third, and fourth cervical vertebrae were traced for cervical staging. Two examiners (A.V and R.A) independently determined the CS stages using the method described by Baccetti et al. [13]. Then, the respective lateral cephalograms were reanalyzed by the same examiners after 6–8 weeks. Using Kappa statistics, the interexaminar agreement was found to be 90.0% and intraexaminer were 91.2% and 91.9% for A.V and R.A respectively with a statistically significant (*p* = 0.001) agreement.

On the same day, blood samples were collected by venipuncture, between 9:00 h and noon. In our study, the protocol for phlebotomy—(the drawing of blood)—was adapted from the World Health Organization (WHO) guidelines [27]. About 3 ml of blood was drawn using the closed blood sampling system by the use of a hypodermic needle and syringe technique from a peripheral vein preferably median cubital vein in the antecubital area. Blood was transferred to the sterile blood collection tubes coated with a clot activator and placed in a leak-proof transporter. Each of the samples was given random numbers and sent to the laboratory. Once received in the laboratory, the samples were allowed to clot at room temperature for 1 h after which they were centrifuged for 10 min at approximately 1000 rpm for separation of serum. Using the clean pipette technique, clearly defined separation of serum was aliquoted by disposable pipette tips (1000 μL) into plastic Eppendorf disposable tubes. Serum samples were then labeled and stored in a freezer at − 80 °C. Serum IGF-1 and DHEA-S levels were assessed by enzyme-linked immunosorbent assay test (human IGF-1 and human DHEA-S ELISA Kit, SRB, Shanghai, China).

## Statistical analysis

Data collected was prepared to analyze in the SPSS software (Statistical Package for the Social Sciences, version.25, IBM). Shapiro-Wilk test was applied to test the normality. Based on the distribution, further descriptive statistics were used. The unpaired *t* test was used to compare the mean difference between two groups, and within the group comparison was done by using one-way ANOVA followed by LSD post hoc test. Univariate regression analysis was done to evaluate the correlation between the hormones and the CS stages. The *p* value of < 0.05 was set as statistically significant.

## Results

The data was found to be normally distributed; thus, an unpaired *t* test was used to compare the mean serum hormone levels between the two groups in each CS stage (Table 1). Serum DHEA-S levels were significantly higher in all the CS stages of group A except CS 2 and CS 3 (*p* = 0.001 and 0.0001), whereas serum IGF-1 was found to be significantly higher in all CS stages except CS 1 and CS 3 (*p* = 0.0001). The peak of both serum hormone levels were found to be in CS 4 in group A and CS 3 in group B. The highest mean ± SD serum DHEA-S level was 685.33 ± 39.11 nmol/ml in group A and 578.12 ± 13.76 nmol/ml in group B. The highest mean ± SD serum IGF-1 level was 538.32 ± 32.31 ng/ml in group A and 460.38 ± 13.49 ng/ml in group B.

**Table 1:** Comparison of mean and standard deviation of IGF-1 (ng/ml) and DHEA-S (nmol/ml) levels among CS stages between group A and group B

CS stages (***n*** = M, F)	Hormone	Group A (male)	Group B (female)	***p*** value
		Mean ± SD	Mean ± SD	
**CS 1 (*n*= 8, 8)**	IGF-1	164.22 ± 12.36	197.19 ± 8.99	0.000***
	**DHEA-S**	**232.17 ± 15.20**	**185.03 ± 18.13**	0.000***
**CS 2 (*n*= 8, 8)**	IGF-1	256.06 ± 15.04	232.50 ± 9.79	0.002**
	**DHEA-S**	**295.40 ± 14.22**	**344.91 ± 30.77**	0.001**
**CS 3 (*n*= 7, 7)**	IGF-1	332.31 ± 16.27	460.38 ± 13.49	0.000***
	**DHEA-S**	**451.77 ± 34.20**	**578.12 ± 13.76**	0.000***
**CS 4 (*n*= 7, 7)**	IGF-1	538.32 ± 32.31	228.33 ± 8.96	0.000***
	**DHEA-S**	**685.33 ± 39.11**	**308.32 ± 7.68**	0.000***
**CS 5 (*n*= 7, 7)**	IGF-1	212.76 ± 4.01	206.31 ± 6.77	0.050*
	**DHEA-S**	**351.13 ± 29.80**	**229.70 ± 17.82**	0.000***
**CS 6 (*n*= 8, 8)**	IGF-1	184.38 ± 12.84	175.27 ± 6.12	0.920^ns^
	**DHEA-S**	**253.38 ± 22.14**	**216.98 ± 15.44**	0.002**

*p* value is significant at the 0.05 level

*ns* non-significant

*p < 0.05, **p < 0.01, ***p < 0.001

ANOVA results showed that there was a significant difference in the serum hormone level among the different CS stages in both the groups for both the hormones (Table 2). In group A, the LSD post hoc test revealed that there was a significant difference in the serum IGF-1 levels in between each CS stages (Table 3). The change in the hormone levels was a significant increase from the CS 1 to CS 2 (*p* < 0.001), then CS 2 to CS 3 (*p* < 0.001), from CS 3 to CS 4 (*p* < 0.001), thereafter decrease till CS 6 (*p* < 0.001). The same pattern was also found in the DHEA-S hormone levels (Table 3). In group B, IGF-1 hormone levels increased significantly from the CS 1 till CS 3 (*p* < 0.001) to the highest and then significantly decreased till the CS 6 (Table 4). DHEA-S hormone levels followed a similar pattern with significant changes except in stages CS 5 and CS 6 (Table 4).

**Table 2:** Comparison of serum hormone levels within the groups among the CS stages (ANOVA)

Hormone	CS 1	CS 2	CS 3	CS 4	CS 5	CS 6	*p* value
IGF-1 (group A)	164.22 ± 12.36	256.06 ± 15.04	332.31 ± 16.27	538.32 ± 32.31	212.76 ± 4.01	184.38 ± 12.84	0.000***
DHEA-S (group A)	**232.17 ± 15.20**	**295.40 ± 14.22**	**451.77 ± 34.20**	**685.33 ± 39.11**	**351.13 ± 29.80**	**253.38 ± 22.14**	0.000***
IGF-1 (group B)	197.19 ± 8.99	232.50 ± 9.79	460.38 ± 13.49	228.33 ± 8.96	206.31 ± 6.77	175.27 ± 6.12	0.000***
DHEA-S (group B)	**185.03 ± 18.13**	**344.91 ± 30.77**	**578.12 ± 13.76**	**308.32 ± 7.68**	**229.70 ± 17.82**	**216.98 ± 15.44**	0.000***

*p* value is significant at the 0.05 level

*ns* non-significant

*p < 0.05, **p < 0.01, ***p < 0.001

**Table 3:** Comparison of IGF-1 and DHEA-S levels in between the CS stages using the LSD test in group A

Dependent variable	(I) CS stage	(J) CS stage	Mean difference (I-J)	Std. error	Sig.	95% confidence interval	
						Lower bound	Upper bound
IGF-1	1	2	− 91.84	8.68	0.000***	− 109.40	− 74.27
	2	3	− 76.24	8.98	0.000***	− 94.42	− 58.06
	3	4	− 206.01	9.28	0.000***	− 224.79	− 187.24
	4	5	325.56	9.28	0.000***	306.78	344.33
	5	6	28.38	8.98	0.003***	10.20	46.56
	6	1	20.15	8.68	0.026*	2.59	37.71
DHEA-S	1	2	− 63.236	13.39	0.000***	− 90.33	− 36.13
	2	3	− 156.366	13.86	0.000***	− 184.41	− 128.31
	3	4	− 233.559	14.32	0.000***	− 262.52	− 204.59
	4	5	334.198	14.32	0.000***	305.23	363.16
	5	6	97.745	13.86	0.000***	69.69	125.79
	6	1	21.217	13.39	0.121^ns^	− 5.87	48.31

The mean difference is significant at the 0.05 level

*ns* non-significant

*p < 0.05, **p < 0.01, ***p < 0.001

**Table 4:** Comparison of IGF-1 and DHEA-S levels in between the CS stages using the LSD test in group B

Dependent variable	(I) CS stage	(J) CS stage	Mean difference (I-J)	Std. error	Sig.	95% confidence interval	
						Lower bound	Upper bound
IGF-1	1	2	− 35.31	4.63	0.000***	− 44.69	− 25.94
	2	3	− 227.87	4.79	0.000***	− 237.57	− 218.17
	3	4	232.04	4.95	0.000***	222.02	242.06
	4	5	22.02	4.95	0.000***	12.00	32.04
	5	6	31.03	4.79	0.003***	21.33	40.74
	6	1	− 21.91	4.63	0.026*	− 31.28	− 12.54
DHEA-S	1	2	− 159.87	9.47	0.000***	− 179.03	− 140.71
	2	3	− 233.21	9.80	0.000***	− 253.05	− 213.38
	3	4	269.80	10.12	0.000***	249.32	290.29
	4	5	78.61	10.12	0.000***	58.13	99.09
	5	6	12.72	9.80	0.202^ns^	− 7.10	32.55
	6	1	31.94	9.47	0.002**	12.78	51.10

The mean difference is significant at the 0.05 level

*ns* non-significant

*p < 0.05, **p < 0.01, ***p < 0.001

Table 5 summarized the comparison of mean and standard deviation of BMI in different CS stages between group A and group B. Table 6 outlines the results of univariate regression analysis. When the CS stages were combined as pre-pubertal (CS 1 and 2), pubertal (CS 3 and 4), and post-pubertal (CS 5 and 6) and analyzed, IGF-1 hormone levels are more likely to increase in pubertal stages compared to pre-pubertal stages (OR = 1.010); also, DHEA-S levels were more likely to hike in pubertal stages compared to pre-pubertal stages (OR = 1.004) and both were statistically significant (Table 6).

**Table 5:** Comparison of mean and standard deviation of BMI in different CS stages between group A and group B

CS stages (*n* = M, F)	Group A (male)	Group B (female)	*p* value
	Mean ± SD	Mean ± SD	
Stage 1 (*n* = 8, 8)	17.9 ± 1.7	17.2 ± 1.4	0.40^ns^
Stage 2 (*n* = 8, 8)	19.3 ± 1.0	20.1 ± 1.2	0.17^ns^
Stage 3 (*n* = 7, 7)	17.8 ± 1.6	20.1 ± 1.4	0.019*
Stage 4 (*n* = 7, 7)	21.4 ± 0.9	19.0 ± 1.1	0.001**
Stage 5 (*n* = 7, 7)	23.3 ± 2.0	20.7 ± 1.9	0.035*
Stage 6 (*n* = 8, 8)	22.3 ± 2.2	21.3 ± 1.5	0.29^ns^

*p* value is significant at the 0.05 level

*ns* non-significant

*p < 0.05, **p < 0.01, ***p < 0.001

**Table 6:** Univariate regression analysis of relationship of IGF-1 and DHEA-S with CS stages

Parameter estimates									
CS stages		B	Std. error	Wald	Df	Sig.	Exp (B)	95% confidence interval for Exp (B)	
								Lower bound	Upper bound
Pubertal	**DHEA**	0.004	0.002	5.517	1	0.019*	1.004	1.001	1.007
	IGF	0.010	0.003	8.628	1	0.003**	1.010	1.003	1.016
Post-pubertal	**DHEA**	− 0.006	0.003	4.691	1	0.030*	0.994	0.988	0.999
	IGF	− 0.012	0.006	4.884	1	0.027*	0.988	0.977	0.999

The reference category is pre-pubertal

*p* value is significant at the 0.05 level

ns non-significant

*p < 0.05, **p < 0.01, ***p < 0.001

## Discussion

The skeletal deficiencies are intercepted and treated well when the intervention is given at the right time, which focuses mainly on the pubertal growth spurt. The goal of the present study was to evaluate whether serum DHEA-S could be used as a biomarker for assessing skeletal maturation during the pubertal growth spurt. Based on the cervical stages of Baccetti et al. [13] and cervical vertebrae maturation indicator, the samples were divided among the two groups. Thus, the relationship between serum DHEA-S levels and the CS stages could be used as a possible predictor for the assessment of pubertal growth spurt. In our study, we have taken a parallel comparator, i.e., serum IGF-1 levels, which is an established biomarker for the same purpose. Thus, the serum IGF-1 levels were measured in each stage, and its change in levels with each CS stage was compared to DHEA-S level, which would reassure the obtained results.

Among the CS stages, the highest serum DHEA-S levels were observed in the CS 3 in group B and CS 4 in group A, and the values were 578.12 ± 13.76 nmol/ml and 685.33 ± 39.11 nmol/ml respectively (Table 1). This was in contrast with the study done by Srinivasan and Premkumar [28]. In their study, subjects were divided as pre-pubertal, pubertal, and adult groups based on the hand-wrist radiographs and the sample consisted of subjects with age ranges from 7 to 30. The highest mean ± SD serum hormone level was observed in the adult group. As supported by Kroboth et al. study [24], the DHEA-S levels peak around 20–30 years of age then decreases gradually, which would be the reason for this difference in both the studies. In our study, maximum age limit taken was 21 years and grouping was focused mainly on the CS stages which are clinically related to the maxillary and mandibular growth. Though the previous study found a significant difference between pre-pubertal, pubertal, and adult groups, they were not in respect with CS stages from lateral cephalogram. The peak value observed in our study was in consistent with the first chronological peak DHEA-S values observed in the previous study [29].

Mean serum hormone levels were found to be least in the CS 6 in females and CS 1 in the males. But from the CS 1 stage, there was a significant gradual increase in the hormone levels in each CS stage until it reaches the highest value in respective groups. After reaching the peak value, there was a decrease in the serum levels. In group A, a decrease was found from CS 4 to CS 5 and to CS 6, whereas in group B, from CS 3 to CS 4, CS 4 to CS 5, and to CS 6. The peak values of serum hormones observed in the CS stages of respective groups are consistent with the pubertal growth spurts of the respective groups, i.e., CS 3 in females and CS 4 in males [13]. When accounting the sexual dimorphism, significant difference was found between the males and females in the DHEA-S levels. This could be due to the influence and interaction of difference in the sexual maturity, difference in the BMI (Table 5), and the growth pattern in the males and females than gender difference itself [30–32].

**Fig. 1:**
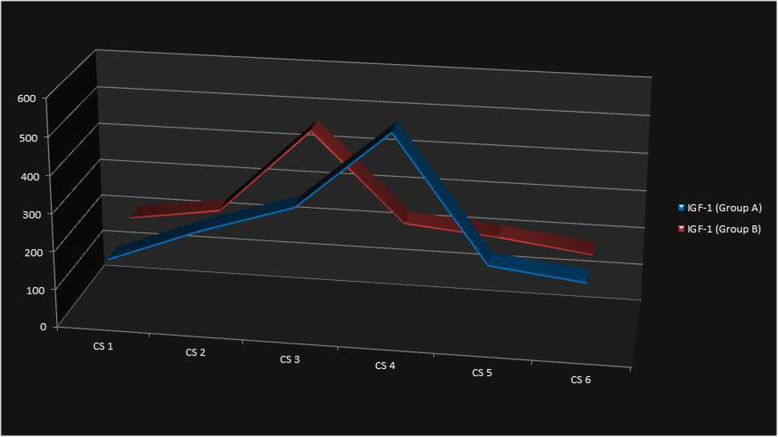
Serum IGF-1 levels in each CS stage in group A and group B

**Fig. 2:**
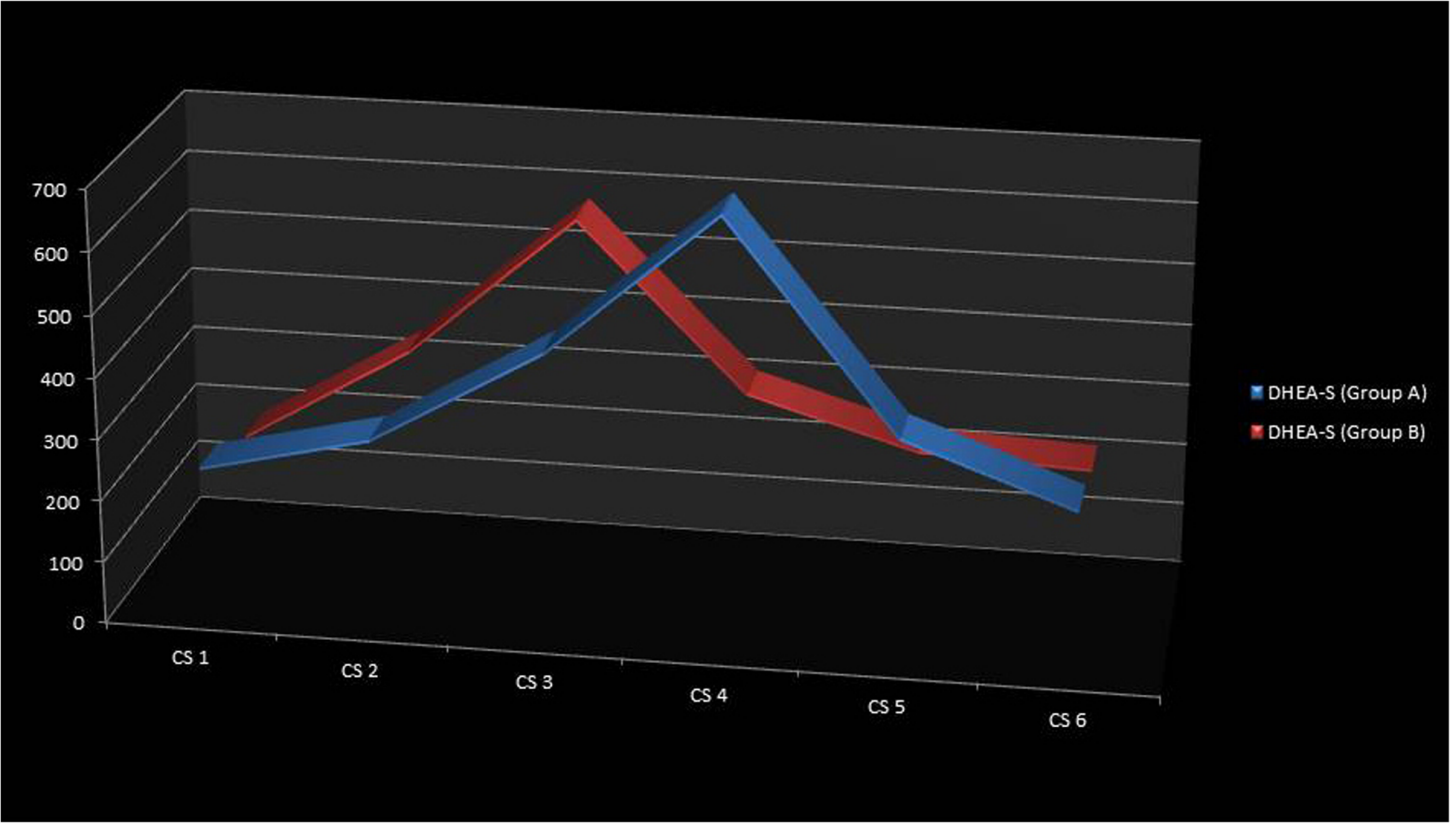
Serum DHEA-S levels in each CS stage in group A and group B

While observing the serum IGF-1 levels, the highest mean serum IGF-1 level was found to be in CS 4 in group A and CS 3 in group B, with values of 538.32 ± 32.31 ng/ml and 460.38 ± 13.49 ng/ml respectively, with a significant sexual dimorphism observed in each CS stage. This was consistent with the results of the previous studies [14, 18], but the values were in different ranges from the previous study which could be due to difference in the ethnicity of the subjects studied. People from different ethnicity have a different range of normal serum IGF-1 hormone levels. The change in IGF-1 values in each stage followed a typical pattern which was also found in the serum DHEA-S levels (Fig. 1 and Fig. 2). In addition, the difference between the hormone levels in each CS stage was statistically significant with a clear interval in both groups. A univariate regression analysis was done after combining the CS 1 and CS 2 as pre-pubertal, CS 3 and CS 4 as pubertal, and CS 5 and CS 6 as post-pubertal (Table 6). Compared to the pre-pubertal stage values, the pubertal stage was more likely to be predicted by both the hormones and it was statistically significant (*p* < 0.05) with the odds ratio that was almost same for both the hormones. Thus, it supported the use of the serum DHEA-S hormone levels to predict the different circumpubertal stages.

The serum DHEA-S levels followed a pattern similar to the pubertal growth curve and to the serum IGF-1 level. The peak in the graph was consistent with the pubertal-skeletal growth accelerations. In addition, the regression analysis revealed the possible association with the different stages. But the only difference was the comparable difference found in the serum DHEA-S levels between the CS 5 and CS 6 in group B which may reduce the use of DHEA-S levels to assess the CS 5 and CS 6 stages. Otherwise, the findings support the definitive relationship between the serum DHEA-S levels and the CS stages. Hence, this relationship between the serum DHEA-S and the CS stages among the circumpubertal age rejected the null hypothesis. The preliminary work done with a relatively small sample size in a cross-sectional study design could be a possible set back of our study, which needs further prospective analysis in a larger population.

## Conclusion

1. The peak serum DHEA-S level was found in CS 3 stage in females and in CS 4 in males with values 578.12 ± 13.76 nmol/ml and 685.33 ± 39.11 nmol/ml respectively.
2. There was a significant difference in the mean serum DHEA-S levels in between each CS stages of both the groups except CS 5 and CS 6 in the females.
3. A definite relationship was found between the serum DHEA-S levels and the CS stages from the lateral cephalogram.
4. The mean serum DHEA-S levels followed a typical pattern from the CS 1 till CS 6 which was comparable and similar with the mean serum IGF-1 levels and pubertal growth curve.
5. Thus, serum DHEA-S levels could be used as a possible diagnostic test for the assessment of the pubertal growth spurt in dentofacial orthopedics.

## Data Availability

As a corresponding author, I have full access to all the data of this study and final responsibility for the decision to submit for publication

## Abbreviations

DHEA-S: Dehydroepiandrosterone-sulfate
IGF-1: Insulin-like growth factor-1
CS: Cervical stages
CVM stage: Cervical vertebrae maturation stage
IAP: Indian Association of Paediatrics
BMI: Body mass index
ELISA: Enzyme-linked immunosorbent assay
ANOVA: Analysis of variance
LSD post hoc: Least significant difference post hoc test
SPSS: Statistical Package for the Social Sciences
